# Endophytic Fungi Associated with Coffee Leaves in China Exhibited In Vitro Antagonism against Fungal and Bacterial Pathogens

**DOI:** 10.3390/jof8070698

**Published:** 2022-06-30

**Authors:** Li Lu, Samantha C. Karunarathna, Kevin D. Hyde, Nakarin Suwannarach, Abdallah M. Elgorban, Steven L. Stephenson, Salim Al-Rejaie, Ruvishika S. Jayawardena, Saowaluck Tibpromma

**Affiliations:** 1 Center for Yunnan Plateau Biological Resources Protection and Utilization, Yunnan Engineering Research Center of Fruit Wine, College of Biological Resource and Food Engineering, Qujing Normal University, Qujing 655011, China; 6371105004@lamduan.mfu.ac.th (L.L.); samanthakarunarathna@gmail.com (S.C.K.); 2 Center of Excellence in Fungal Research, Mae Fah Luang University, Chiang Rai 57100, Thailand; kdhyde3@gmail.com (K.D.H.); ruvi.jaya@yahoo.com (R.S.J.); 3 School of Science, Mae Fah Luang University, Chiang Rai 57100, Thailand; 4 Innovative Institute for Plant Health, Zhong Kai University, Guangzhou 510550, China; 5 Research Center of Microbial Diversity and Sustainable Utilization, Faculty of Science, Chiang Mai University, Chiang Mai 50200, Thailand; suwan_461@hotmail.com; 6 Department of Botany and Microbiology, College of Science, King Saud University, Riyadh P.O. Box 145111, Saudi Arabia; aelgorban@ksu.edu.sa; 7 Department of Biological Sciences, University of Arkansas, Fayetteville, AR 72701, USA; slsteph@uark.edu; 8 Department of Pharmacology & Toxicology, College of Pharmacy, King Saud University, Riyadh P.O. Box 145111, Saudi Arabia; rajaie@ksu.edu.sa

**Keywords:** biocontrol agents, *Coffea*, coffee-endophytic fungi, pathogenic fungi, pathogenic bacteria

## Abstract

Coffee endophytes have been studied for almost 74 years, and several studies have demonstrated coffee-endophytic fungi with antibacterial and antifungal potential for human and plant pathogens. In this study, we isolated and identified a total of 235 strains of endophytic fungi from coffee leaf tissues collected in four coffee plantations in Pu’er city, Yunnan province, China. Molecular identification was carried out using maximum likelihood phylogenetic analysis of nuclear ribosomal internal transcribed spacer (ITS1-5.8S rDNA-ITS2) sequences, while the colonization rate and the isolation frequency were also calculated. Two pathogenic fungi (*Alternaria alternata* and *Penicillium digitatum*) and two pathogenic bacteria (*Pseudomonas syringae* and *Salmonella enterica* subsp. *enterica*) were used for screening the antagonistic activities of 61 strains of coffee-endophytic fungi by a dual-culture test assay while maximum likelihood phylogenetic analysis confirmed their natural classification. This is the first study of coffee-leaf-endophytic fungal diversity in China, and the results revealed that coffee-endophytic fungi from this study belong to the Ascomycota, distributed among two classes, 10 orders, and 17 families. Concurrently, endophytic fungi isolates distributed in *Arthrinium*, *Biscogniauxia*, *Daldinia*, *Diaporthe,* and *Nigrospora* showed strong antagonistic activities against the pathogens. For the pathogens *Alternaria alternata* and *Pseudomonas syringae*, *Nigrospora* XCE-7 showed the best inhibitory effects with inhibition rates of 71.76% and 61.11%, respectively. For the pathogen *Penicillium digitatum*, *Daldinia* ME-9 showed the best inhibitory effect with a 74.67% inhibition rate, while *Biscogniauxia* PTE-7 and *Daldinia* T5E-1-3 showed the best inhibitory effect with a rate of 60.42% against the pathogen *Salmonella enterica* subsp. *enterica*. Overall, our study shows the diversity of coffee endophytes in four coffee-growing areas in Pu’er city, Yunnan province, China, and their potential use as biological control agents against two fungal and two bacterial pathogens.

## 1. Introduction

Coffee is one of the most important crops around the world that grow in tropical and subtropical areas, and it is the second-largest export commodity after crude oil [1,2]. Coffee is susceptible to diseases and pests, but most coffee diseases are caused by pathogenic fungi, which can infect various tissues of coffee before and after coffee harvest and affect the yield and quality of the fruit [3,4]. Cerda et al. [5] showed that pests and diseases cause up to 38% of coffee yield losses. In order to reduce the impact of diseases on coffee, cultural control, chemical control, and biological control strategies have been developed [6,7]. Cultural control can avoid and reduce some plant diseases and generally requires workers to implement it manually, which is time-consuming and labor-intensive [7]. Chemicals can control diseases and increase yields, such as the use of the fungicides cyproconazole and thiamethoxam in the soil to control rust disease [8], but the chemical methods can result in environmental and food contamination, which forces society to use nonchemical methods for plant disease control [9]. Carl von Tubeuf was the first person to use biological control to manage plant diseases in 1914 [10,11,12,13]. The biological control of plant diseases occurs with several distinct mechanisms, including competition for nutrients between a pathogen and a harmless species, parasitism, and production of antibiotics [14,15]. Biological controls have the advantages of being environmentally compatible, can exhibit broad or narrow targets depending on the organism, can be site-specific, less prone to resistance, safe, and inexpensive compared with chemical pesticides. Due to these advantages, biocontrol has become a popular method of plant disease management [16,17,18]. Currently, some fungi and bacteria as biocontrol agents are available as commercial products [17]. A total of 101 microbial biological control agents were registered for disease control in Australia, Brazil, Canada, Europe, Japan, New Zealand, and the United States in 2017 [19].

Endophytes are microorganisms such as actinomycetes, bacteria, or fungi that live in plant tissues and do not cause the host to produce any disease symptoms [20,21,22]. They is generally considered reciprocal and neutral, but can cause disease or become saprophytic when senescence occurs in the host, its vitality decreases, and other factors are weakened [23,24]. The host strongly restricts the growth of endophytes, but it can overcome the host’s defense by secreting biologically active metabolites or changing the balance of plant hormones in the host [24,25,26]. Endophytes spend all or part of their life cycle in the healthy tissues of plants [27,28]. The bioactive compounds synthesized by endophytes enhance the resistance of plants to pathogenic microorganisms and pests, protect the host from environmental stresses such as high temperature and drought, and help the host to more easily access nutrients to promote the growth necessary for a stable symbiosis [21,26,29]. These bioactive metabolites produced by endophytes can be used directly or indirectly as potential biological fertilizers to promote plant growth and biocontrol agents to control pathogenic-causing diseases [28,30,31,32,33]. Fadiji and Babalola [34] summarized the applications of endophytes into four categories. These are: (i) plant growth promotion, including nutrient uptake, photostimulation, and phosphate solubilization; (ii) plant health and protection (antimicrobial activities and pathogen displacement); (iii) pollution control and phytoremediation; and (iv) medical and industrial applications (anticancer and antiviral). Since endophytes can produce large amounts of biologically active substances, the use of endophytes to control plant diseases and to increase crop yields has become the focus of the agricultural industry [17,35,36,37].

Endophytic fungi are one of the groups of microorganisms that colonize plant tissues and interact with plants, which can be used for biological control and resistance induction, and these fungi are one of the most interesting groups with high utilization value and high diversity [38]. Endophytic fungi are known to produce important secondary metabolites such as anticancer, antifungal, antidiabetic, and immunosuppressant compounds, which can be used as a source of some important secondary metabolites [39,40]. Numerous attempts have been made to control wilt diseases through biocontrol agents using endophytic fungi, and this way holds great promise for improving crop productivity while serving as an alternative to chemical fungicides and synthetic fertilizers [41,42,43,44]. A huge number of studies have shown that fungal endophytes have potential research values (e.g., endophytic fungi secrete hormones such as indoleacetic acid and gibberellins that aid in plant developmental processes and improve plant growth and crop productivity) [45,46]. The endophytic fungus *Penicillium indica* can regulate the production of phytohormones and contributes to root growth in crops such as barley and tobacco [47,48]. The endophytic fungus *Acremonium alternatum* controls the damage caused by the moth *Plutella xylostella* in beans and induces resistance against *Leveillula taurica* (the powdery mildew pathogen) in tomatoes [49]. Ek-Ramos et al. [49] showed that the cotton fungal endophyte *Phomopsis* sp. reduces caterpillar herbivory activity in cotton plants. The endophytic fungi *Aspergillus terreus* and *Penicillium citrinum* reduce the pathogenic *Sclerotium rolfsii* and induce increases in the biomass yield of sunflower plants [50]. Many studies have revealed that endophytic fungi, such as *Ampelomyces quisqualis*, *Aureobasidium pullulans*, *Beauveria bassiana*, *Botryosphaeria* sp., *Candida oleophila*, *Coniothyrium minitans*, *Gliocladium catenulatum*, *Metarhizium anisopliae*, *Paecilomyces lilacinus*, *Pestalotiopsis microspora*, *Phlebiopsis gigantea*, *Purpureocillium lilacinum*, *Trichoderma asperellum*, *T. atroviride*, *T. gamsii*, *T. polysporum,* and *Verticillium lecanii,* can be used as biocontrol agents in commercial products [51,52,53,54,55,56]. Therefore, endophytic fungi represent an effective agent in managing diseases and promoting plant growth; they also provide great opportunities for researching new products.

Studies of endophytic fungi in coffee were pioneered by Rayner in 1948 [57]. Since 1948, many studies have been published on coffee endophytes but no research has been carried out on the antagonistic effects of coffee endophytes as evaluated by a dual-culture assay. *Beauveria bassiana* can be established in coffee seedlings through different inoculation methods and the plants can resist coffee berry borers [58]. Fernandes et al. [59] showed that the fungal endophytes isolated from the healthy leaves of *Coffea arabica* could produce bacteriostatic, antifungal, and antitumor bioactive substances. *Trichoderma* sp. isolated from healthy coffee roots by Mulaw et al. [60] was demonstrated to have an antagonistic effect on the fungus *Fusarium* sp., which causes vascular wilt diseases. Monteiro et al. [61] isolated endophytes from *Coffea arabica*, and the results showed that *Acremonium*, *Muscodor*, and *Simplicillium* have antibacterial activities against *Aspergillus ochraceus*, while six different endophytic fungal strains can inhibit the growth of *Fusarium verticillioides*. The endophyte *Simplicillium coffeanum* isolated from coffee branches by Gomes et al. [62] has antimicrobial activities against *Aspergillus niger*, *A*. *ochraceus*, *A*. *sydowii*, and *A*. *tubingensis*. *Trichoderma harzianum* (CRF1) and *T. viride* (CRF-2), isolated from coffee rhizosphere soil by Ranjini et al. [63], can control *Myrothecium roridum* in vivo, a fungus that causes stem necrosis and leaf spot of coffee seedlings.

Both bacteria and fungi cause diseases in coffee [7]. *Alternaria alternata* was identified in Brazil in 1999 as the causative agent of necrotic spots on coffee leaves [64]. This pathogen is more serious when present in other fruits and vegetables, and the current control methods include fungicides, synthetic chemical fungicides, and biological control [65]. *Penicillium digitatum* is a destructive postharvest pathogen causing green mold in fruit, especially citrus fruits [66]. Fungicides are still the main control agent of postharvest green mold, and it is necessary to develop novel and safer strategies for effectively controlling plant diseases [67]. Bacterial halo blight disease caused by *Pseudomonas syringae* has affected coffee plantations in Brazil and Ethiopia. Due to only a few efficient commercial products being available on the market to control this disease and the low efficiency of the chemical control on field conditions, biological control and disease-resistant variety development should be the most promising management methods [68,69,70]. *Salmonella enterica* subsp. *enterica* is a common pathogen of animals, humans, or plants [71]. *Salmonella* can adhere to plant surfaces before actively infecting the interior of different plants, leading to the colonization of plant organs [72,73] and suppression of the plant immune system [74]. In addition, *Salmonella* originating from plants reduces its virulence toward animals [75]. Therefore, plants act as an alternative host for *Salmonella* pathogens and have a role in transmitting back to animals. Moreover, *Salmonella* can adapt to survive in different environments outside the host organism, such as low pH or high temperatures [76].

The microfungal diversity of the Greater Mekong Subregion (GMS) has been relatively well-studied, but their secondary metabolites have not been well-studied [77]. Since endophytic fungi have become the source of new antibacterial and antifungal agents, with some examples producing valuable secondary metabolites, this study was planned to identify and examine the antagonistic abilities against two fungal and two bacterial pathogens of endophytic fungi, which were isolated from healthy coffee leaves from four coffee-growing areas in Pu’er city, Yunnan province, China.

## 2. Materials and Methods

### 2.1. Sampling Location and Leaf Collection

**Figure 1:**
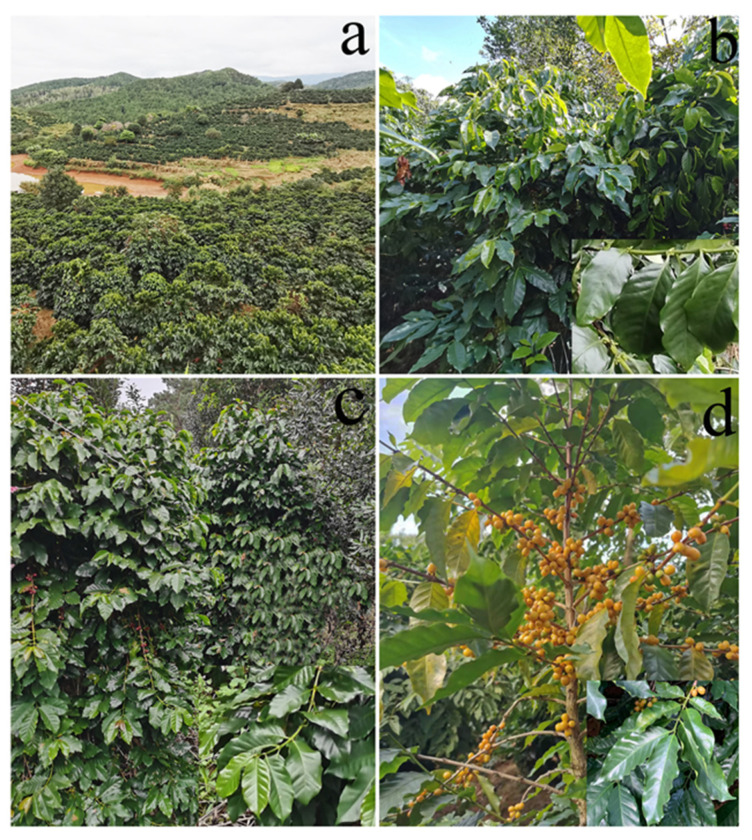
(**a**) Aerial view of a coffee plantation in Pu’er city; (**b**) Arabica coffee trees; (**c**) Catimor coffee trees; (**d**) Yellow Bourbon coffee trees.

Pu’er city has the largest coffee planting area, with the highest yield, and the best-quality coffee production in Yunnan province, China [78]. This study was conducted on mature and healthy leaf samples collected from four coffee-planting areas in Pu’er city, Yunnan province, China (Figure 1, Table 1). All sites were >30 km and <210 km apart. More than 50 healthy leaves were collected from each coffee plant host, placed in clean plastic bags, kept in iceboxes, brought to the laboratory, and refrigerated at 4 °C until further processing.

### 2.2. Coffee Leaves Processing and Fungal Endophytes Isolation

The isolation process of endophytic fungi refers to the slightly modified method of Tibpromma et al. [79] and Du et al. [80]. Sterile water, 70% alcohol, 1% sodium hypochlorite, a sterile scalpel, sterile tissue paper, sterile fine-tip forceps, and potato dextrose agar (PDA) plates (supplemented with amoxicillin) were prepared. Ten healthy leaves were randomly selected for each host and each leaf was washed under running tap water and then air-dried. Working under an aseptic laminar hood, a hole puncher was used to randomly punch the leaves to obtain 5 mm size pieces (10 pieces/leaf) and these were washed in sterile water for one minute, dried on sterile tissue paper, soaked in 70% alcohol for 30 s, transferred to 1% sodium hypochlorite and soaked for one minute, then soaked in 70% alcohol for 30 s, and finally transferred to sterile water for one minute to remove chemicals. Afterwards, they were dried on sterile tissue paper; then, five pieces were placed on 90 mm-diameter PDA plates. A total of 30 leaf pieces were placed on six PDA plates for each host and each location. All PDA plates were incubated at 28 °C and observation of the culture plates was performed every day to check emerging hypha from the edge of the leaves. After colonies had grown, a small piece of mycelium with agar was cut and transferred to new PDA plates to obtain a pure culture [26,81,82]. There was a total of 270 coffee leaf pieces, including Arabica coffee (150 pieces × 5 plants × 1 place), Catimor (90 pieces × 1 plant × 3 places), and Yellow Bourbon (30 pieces × 1 plant × 1 places). A total of 235 pure cultures were isolated in this study (Table 2).

### 2.3. DNA Extraction, PCR Amplification, and ITS Gene Sequencing

The Biospin Fungus Genomic DNA Extraction Kit-BSC14S1 (BioFlux, Beijing, China) was used for DNA extraction from pure cultures grown on PDA for 7–10 days. The extraction process followed the protocol of the manufacturer. Internal transcribed spacer (ITS) regions 1 and 2 of the nuclear ribosomal DNA operon, including the 5.8S region, were amplified by the primers ITS5 and ITS4 [83]. The methods of Lu et al. [84] were followed for the polymerase chain reaction (PCR). Amplification reactions were performed in a 25 μL reaction volume which contained 2 μL DNA, 1 μL of each reverse and forward primers and 8.5 μL ddH2O, 12.5 μL 2 × FastTaq Premix (mixture of FastTaq TM DNA Polymerase, buffer, dNTP Mixture, and stabilizer) (Beijing Qingke Biological Technology Co., Ltd., Beijing, China). The amplified PCR products were sent to Bioer Technology Co., Ltd., Hangzhou, and Beijing Kinco Biotechnology Co., Ltd. Kunming Branch, China for sequencing.

### 2.4. Molecular Identification and Phylogenetic Analyses

A total of 235 sequences were obtained, and the newly generated sequences were merged forward and reversed by Geneious 9.1.2 (https://www.geneious.com/ (Auckland, New Zealand), accessed on 30 June 2022). The BLAST search of the sequences was carried out and the top five high-similarity results were recorded. Among our isolates, resistant genera based on published papers and less frequently isolated genera were selected (61 isolates) for the dual-culture assay. The closest match of sequences for the 61 isolates that were used for dual-culture assay were retrieved from GenBank. Multiple alignments were prepared using the online tool MAFFT version 7 (https://mafft.cbrc.jp/alignment/server/, accessed on 1 June 2022) [85], manually edited in BioEdit v. 7.0.5.2 [86], and then their format converted from FASTA to PHYLIP by ALTER online tool (https://www.sing-group.org/ALTER/, accessed on 1 April 2022) [87]. Phylogenetic analyses of the aligned sequences were conducted using the maximum likelihood method [88]. Maximum likelihood trees were generated via RAxML-HPC version 8 on XSEDE (8.2.12) [89,90] in the CIPRES Science Gateway platform [91] using the GTR+I+G model of evolution. The resulting trees were visualized with FigTree version 1.4.0 and annotated in Microsoft 365 PowerPoint. Sixty-one generated sequences were deposited in GenBank and accession numbers were obtained.

### 2.5. Diversity Analysis

The doughnut chart in Excel 365 was used to show the family, order, and class distribution of endophytic fungi. The colonization rate (CR) and the isolation frequency (IF) of the calculated endophytic fungi: CR = NS/NL × 100%, IF = NS/NT × 100%, NS = number of strains isolated from the location, NL = number of leaf pieces prepared from the location, NT = all number of isolates from all location. Then, the taxonomic diversity based on the species/genera (S/G) ratio was calculated to evaluate the endophyte diversity of each host. In addition, the relative abundances of the species were calculated and the similarity between the fungal communities was estimated using Sorensen’s index [92]. The species diversity of different hosts in each sampling site was calculated by the Shannon diversity (H’), Margalef diversity (d), and the Pielou evenness indices (J’), H’ = -∑ (Pi ln [Pi]), Pi = ni/N, ni = number of individuals of the species i, and N = total number of individuals of all species; d = S-1/Log N, where S is the number of species and N is the total number of specimens in the sample; J’ = H’/Log (S), where H’ is the value obtained by the Shannon index and S is species richness [26,93,94].

### 2.6. Tests for Antagonism: Endophytic Fungi vs. Pathogenic Fungi/Bacteria

**Figure 2:**
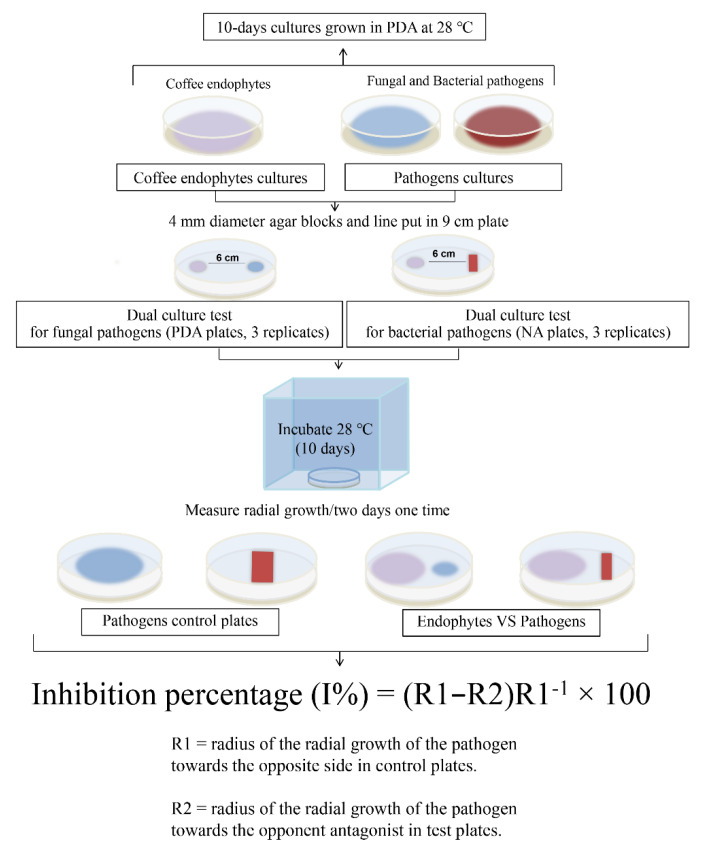
Dual-culture assay steps and the percentage inhibition formula.

Endophytes were selected for dual-culture testing in vitro based on the information reported in the literature [95,96] and the less frequent strains from our results. The living cultures of the 61 endophytic fungi were deposited in the Kunming Institute of Botany Culture Collection (KUMCC). The 61 endophytic fungi were tested for dual-culture assays in 9 cm Petri dishes with two fungal and bacterial pathogens (i.e., *Alternaria alternata*, *Penicillium digitatum*, *Pseudomonas syringae*, and Salmonella enterica subsp. *enterica*) (Table 3) from China General Microbiological Culture Collection Center (CGMCC). These pathogens were selected for our experiments because they are common and cause different diseases on numerous hosts (Table 3) and have been used in biological control tests [97,98,99,100,101,102,103]. The PDA was supplemented with amoxicillin antibiotic and nutrient agar (NA) was prepared without amoxicillin antibiotic. The steps used are given in Figure 2.

## 3. Results

### 3.1. Isolation and Identification of Endophytic Fungi

**Figure 3:**
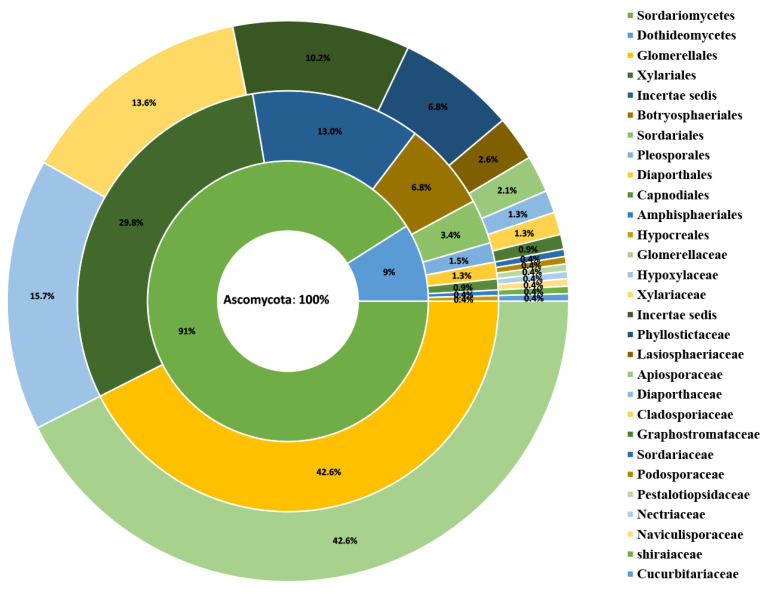
Taxonomic distribution of the fungal taxa (*n =* 235) from the coffee.

A total of 235 fungal endophytes were isolated from 270 coffee leaf pieces obtained from four coffee plantations in Yunnan province, China. All endophytes were used for the molecular identification, based on the blast result of ITS sequences, and the closest species information was obtained. Overall, 235 endophytes belong to Ascomycota (100%) and are distributed among two classes, 10 orders, and 17 families (Figure 3). At the class level, 91% are members of the Sordariomycetes, while 9% are members of the Dothideomycetes. At the order level, the Glomerellales (42.6%) is the largest group, while the Amphisphaeriales (0.4%) and the Hypocreales (0.4%) are reported as the least important. At the family level, the Glomerellaceae (42.6%) is the most common family, while the Cucurbitariaceae (0.4%), Naviculisporaceae (0.4%), Nectriaceae (0.4%), Podosporaceae (0.4%), Pestalotiopsidaceae (0.4%), Sordariaceae (0.4%), and Shiraiaceae (0.4%) are the less important families.

Table 2 shows that 235 species are distributed in 21 genera. *Colletotrichum* is the most common genus, composed of 100 isolates, and found in each location and on each host. This is followed by *Xylaria* (composed of 29 isolates) and *Daldinia* (composed of 25 isolates). Seven genera (*Fusarium*, *Kretzschmaria*, *Naviculispora*, *Neurospora*, *Pestalotiopsis*, *Pyrenochaetopsis*, and *Shiraia*) have a low frequency of occurrence; all of these are represented by one isolate and are distributed in two coffee plantations. In addition, 21 genus names could not be determined from only the blast results of ITS, which indicated these as unidentified fungal endophytes, unidentified fungal species, or an undetermined species in the Sordariomycetes.

### 3.2. Phylogenetic Analyses

**Figure 4:**
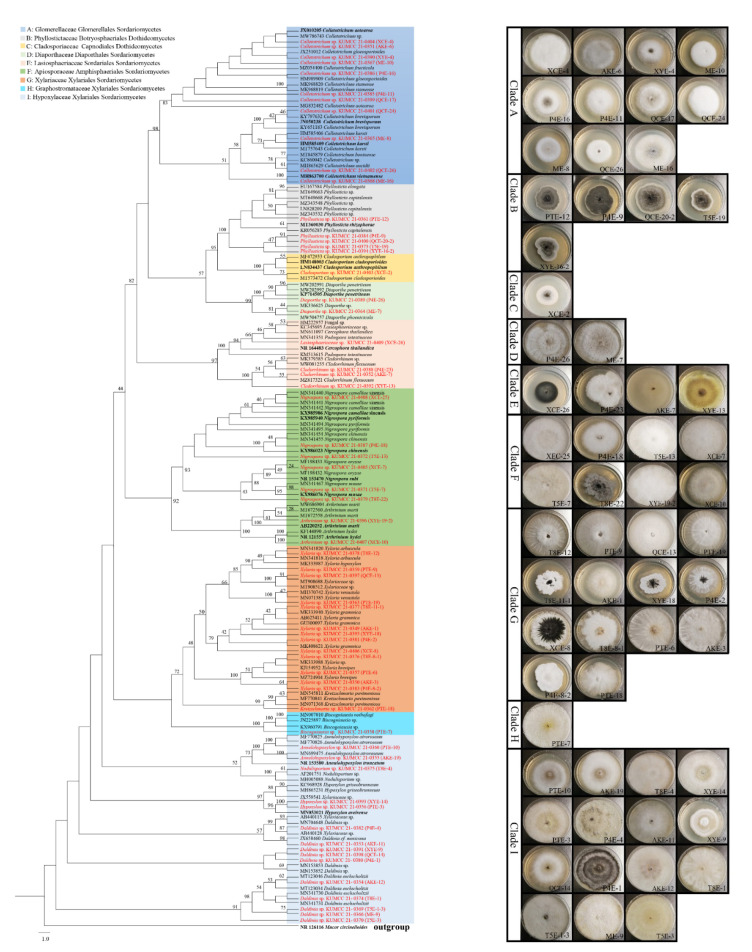
A maximum likelihood analysis showing the phylogenetic relationships of coffee fungal endophytes. The tree has a total of 167 fungal sequences, of which 61 fungal sequences came from the present study and 106 reference sequences of close relatives came from the GenBank. *Mucor circinelloides* (NR126116; Mucoraceae; Mucorale; Mucoromycetes) was used as the outgroup taxon. The bootstrap support values (≥50%) are indicated at the nodes. The bold font indicates the type species from the GenBank and data presented in the red font are the isolate code and culture number of the fungal endophytes obtained from the coffee leaves. Fungal isolates are further highlighted in different colors according to the family classification. Images on the right side of the phylogeny tree are the morphotypes of each clade.

Phylogenetic analyses were carried out on the 61 endophytes that were used for the dual-culture testing. Our ITS sequences were combined with the sequences of close relatives in GenBank to construct an ITS phylogenetic tree that was composed of 167 sequences. The ML analysis of the combined dataset yielded a best scoring tree with a final ML optimization likelihood value of − 13283.937651. The alignment has 684 distinct alignment patterns, with 32.41% completely undetermined characters and gaps. Parameters for the GTR+I+G model: estimated base frequencies A = 0.246497, C = 0.253353, G = 0.238501, T = 0.261649; substitution rates AC = 0.867207, AG = 1.955827, AT = 1.257643, CG = 0.680575, CT = 2.861925, GT = 1.000000; and gamma distribution shape parameter α = 0.259865. The RAxML analysis resulted in a tree which is shown in Figure 4.

The phylogenetic tree includes two classes, seven orders, and nine families. Five orders (Amphisphaeriales, Diaporthales, Glomerellales, Sordariales and Xylariales) and seven families (Apiosporaceae, Diaporthaceae, Glomerellaceae, Graphostromataceae, Hypoxylaceae, Lasiosphaeriaceae and Xylariaceae) belong to the class Sordariomycetes. Another two orders (Botryosphaeriales and Capnodiales) and two families (Cladosporiaceae and Phyllostictaceae) belong to the class Dothideomycetes (Figure 4).

Since the blast result of XCE-26 has a high similarity with both *Podospora intestinacea* (MN341351, 99.40%) and *Cercophora thailandica* (NR_164483, 99.12%), we were not able to determine the genus name and it was treated as Lasiosphaeriaceae sp.

### 3.3. Diversity of Coffee Fungal Endophytes

In Table 4, the highest colonization rate (CR) and the isolation frequency (IF) were reported from *Coffea arabica* (T8667) from the Xiang Yuan Shu He coffee plantation (CR = 106.67%, IF = 13.62%), while Catimor (ME) from the Mo Jiang Jing Gong coffee plantation reported the lowest (CR = 70.00%, IF = 8.94%). By analyzing the taxonomic diversity through the computation of the (S/G) ratio, and the relative abundance of the species through the calculation of Margalef diversity (d), Shannon diversity (H’), and the Pielou evenness indices (J’), we found that *Coffea arabica* (P4) from the Xiang Yuan Shu He coffee plantation showed the highest species richness (d = 5.7407, H’ = 2.8944, J’ = 0.9830), followed by *Coffea arabica* (T5175) from the same coffee plantation (d = 4.7199, H’ = 2.6582, J’ = 0.9587). The number of species and the genera of these two hosts are P4 (18 species, nine genera, S/G = 2.00) and T5175 (15 species, eight genera, S/G = 1.88). Furthermore, the best S/G ratio was reported from *Coffea arabica* (PT) from the Xiang Yuan Shu He coffee plantation, which was reported as 14 species belonging to nine genera, S/G = 1.56.

### 3.4. Dual-Culture Assay

**Figure 5:**
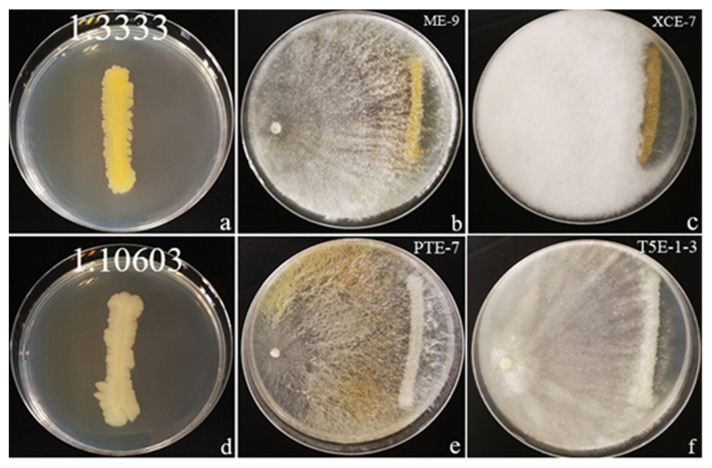
Illustration of an antagonist test by the dual-culture technique with two bacterial pathogens and an inhibition rate greater than or equal to 60%. The two pathogens were cocultivated with the coffee fungal endophytes on NA plates and incubated for 10 days at 28 °C. (**a**) *Pseudomonas syringae* control plate; (**b**,**c**) Coffee endophytes inhibit *Pse. syringae*; (**d**) *Salmonella enterica* subsp. *enterica* control plate; (**e**,**f**) Coffee endophytes inhibit *S. enterica* subsp. *enterica*.

**Figure 6:**
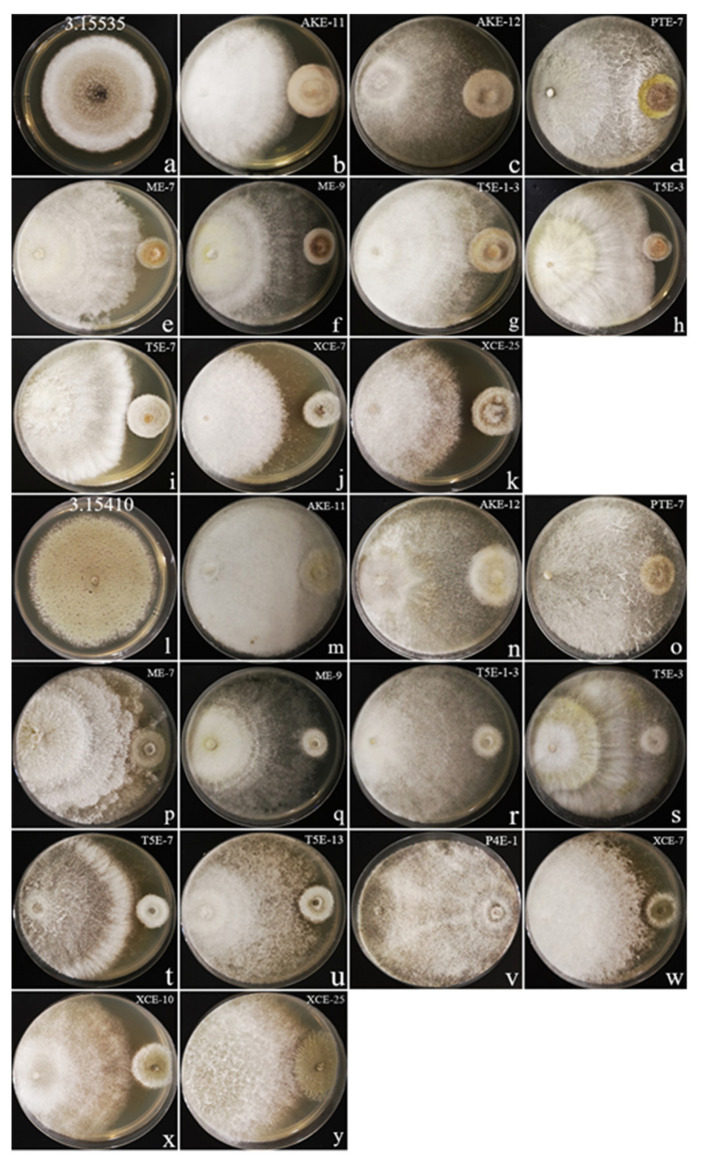
Illustration of an antagonist test using the dual-culture technique with two fungal pathogens and an inhibition rate greater than or equal to 60%. The two pathogens were cocultivated with the coffee fungal endophytes on PDA plates and incubated for ten days at 28 °C. (**a**) *Alternaria alternata* control plate; (**b**–**k**) Coffee endophytes inhibit *A. alternata*; (**l**) *Penicillium digitatum* control plate; (**m**–**y**) Coffee endophytes inhibit *Pen. digitatum*.

To evaluate the antagonistic abilities of coffee endophytes, 61 coffee endophytes along with four pathogens were selected for a dual-culture assay in our study. The results are shown in Table 5. We had some dual-culture plates grown for 10 days with an inhibition rate greater or equal to 60%. For bacterial pathogens, only two endophytes exhibited significant values of growth inhibition against *Pseudomonas syringae* (1.3333) and *Salmonella enterica* subsp. *enterica* (1.10603) (Figure 5). In contrast, the effect of fungal pathogens (Figure 6) was appreciably greater than that of bacteria. For example, 13 endophytes exhibited significant values of growth inhibition against *Alternaria alternata* (3.15535) and 10 endophytes exhibited significant values of growth inhibition against *Penicillium digitatum* (3.15410).

#### 3.4.1. Effect of Coffee Endophytes on the Growth of *Pseudomonas syringae* (1.3333)

*Nigrospora* sp. XCE-7 (Figure 5b) was able to inhibit the growth of *Pse. syringae* with an inhibition rate of 61.11%, and *Daldinia* sp. ME-9 (Figure 5c) showed an inhibition rate of 60.00%. The *Pse*. *syringae* control plate is shown in Figure 5a.

#### 3.4.2. Effect of Coffee Endophytes on the Growth of *Salmonella enterica* subsp. *enterica* (1.10603)

*Biscogniauxia* sp. PTE-7 (Figure 5e) and *Daldinia* sp. T5E-1-3 (Figure 5f) showed the same inhibition rate of 60.42% against the growth of *S*. *enterica* subsp. *enterica*. PTE-7 produced a yellow mycelium after it was in contact with bacterial pathogens. The *S. enterica* subsp. *enterica* control plate is shown in Figure 5d.

#### 3.4.3. Effect of Coffee Endophytes on the Growth of *Alternaria alternata* (3.15535)

*Nigrospora* sp. XCE-7 (Figure 6j) inhibited the growth of *A. alternata* with an interaction characterized by a clear zone around the pathogen and an inhibition rate of 71.76%. It was followed by *Daldinia* sp. ME-9 (Figure 6f) with an inhibition rate of 71.30%. Those demonstrating inhibition rates greater than or equal to 60 were *Daldinia* sp. T5E-3 (Figure 6h, 68.52%), *Nigrospora* sp. T5E-7 (Figure 6i, 65.74%), *Daldinia* sp. T5E-1-3 (Figure 6g, 63.43%), *Diaporthe* sp. ME-7 (Figure 6e, 63.43%), *Nigrospora* sp. XCE-25 (Figure 6k, 62.50%), *Biscogniauxia* sp. PTE-7 (Figure 6d, 61.11%), *Daldinia* sp. AKE-12 (Figure 6c, 60.19%), and *Daldinia* sp. AKE-11 (Figure 6b, 60.00%). The *A. alternata* control plate is shown in Figure 6a.

#### 3.4.4. Effect of Coffee Endophytes on the Growth of *Penicillium digitatum* (3.15410)

*Daldinia* sp. ME-9 (Figure 6q) inhibited the growth of *Pen. digitatum* with an inhibition rate of 74.67%, and this was followed by *Nigrospora* sp. T5E-7 (Figure 6t) with an inhibition rate of 72.00%. Inhibition rates greater than or equal to 60 were found in *Daldinia* sp. T5E-1-3 (Figure 6r, 70.67%), *Daldinia* sp. T5E-3(Figure 6s, 68.00%), *Nigrospora* sp. XCE-7 (Figure 6w, 68.00%), *Daldinia* sp. AKE-11 (Figure 6m, 67.56%), *Biscogniauxia* sp. PTE-7 (Figure 6o, 67.56%), *Nigrospora* sp. XCE-25 (Figure 6y, 67.56%), *Nigrospora* sp. T5E-13 (Figure 6u, 65.78%), *Arthrinium* sp. XCE-10 (Figure 6x, 64.90%), *Diaporthe* sp. ME-7 (Figure 6p, 64.89%), *Daldinia* sp. P4E-1 (Figure 6v, 64.89%), and *Daldinia* sp. AKE-12 (Figure 6n, 64.00%). Among these, XCE-7 and T5E-13 inhibited the growth of *Pen. digitatum* by displaying a small zone around the pathogen. The *Pen*. *digitatum* control plate is shown in Figure 6l.

Overall, this dual-culture assay demonstrated that most coffee endophytes inhibit the growth of pathogens by competition for nutrients and space, while only two endophytes displayed the mechanism of antibiosis to inhibit the growth of pathogens by producing metabolites (antibiotics and enzymes). One of the *Nigrospora* sp. (XCE-7) isolates produced a clear zone of inhibition for *Pseudomonas syringae*, *Alternaria alternata*, and *Penicillium digitatum*, while *Nigrospora* sp. (T5E-13) had a zone of inhibition only for *Pen. digitatum*. In this study, *Arthrinium* sp., *Biscogniauxia* sp., *Daldinia* sp., *Diaporthe* sp. and *Nigrospora* sp. produced different levels of inhibitory effects on the growth of pathogens. *Arthrinium* sp. (XCE-10) inhibited only the growth of one fungal pathogen (*Pen. digitatum*), but *Diaporthe* sp. (ME-7) inhibited the growth of two fungal pathogens (*Alternaria alternata* and *Pen. digitatum*). *Biscogniauxia* sp. (PTE-7), *Daldinia* sp. (AKE-11, AKE-12, ME-9, P4E-1, T5E-1-3, and T5E-3), and *Nigrospora* sp. (T5E-13, T5E-7, XCE-7, and XCE-25) inhibited the growth of fungal pathogens as well as bacterial pathogens.

## 4. Discussion

The results of our study agree with the results of previous studies [59,81,93,104], which showed that *Colletotrichum* and *Xylaria* are the dominant endophytes in coffee leaves. However, the species richness and diversity of endophytes as determined in the present study are different and higher than those reported by Santamarıá and Baymanet [81] in Puerto Rico, Saucedo-García et al. [104] in Mexico, and Oliveira et al. [93] in Brazil for coffee leaf endophytes. Species of *Colletotrichum* are common saprobes, pathogens, and endophytes on a range of economically important plant hosts in tropical regions [105,106], and species of *Xylaria* are recognized as saprotrophic fungi and as endophytic fungi of many plants [107]. Other unique and less frequently encountered species were reported, most likely due to different climates, altitude, humidity, and sampling period. Herein, nine genera are reported for the first time to be isolated as coffee endophytes. These are *Annulohypoxylon*, *Biscogniauxia*, *Kretzschmaria*, *Naviculispora*, *Neurospora*, and *Pyrenochaetopsis*, and they were isolated from *Coffea arabica* in the Xiang Yuan Shu He coffee plantation. *Fusarium* sp. and *Shiraia* sp. were isolated from Catimor in the Qi Xiang coffee plantation, and *Nemania* sp. was isolated from Yellow Bourbon in the Xiao Ao Zi coffee plantation. In this study, only the PDA medium was used, and it is possible that some species were not capable of growing on PDA.

Endophytic fungi isolated from *Coffea* showed promising sources of bioactive compounds [61]. Fulthorpe et al. [108] reported the isolation of coffee root endophytes that have potential ecological roles with *Coffea arabica* based on next-generation sequencing, and *Burkholderia*, *Enterobacter* and *Pantoea* were reported as the dominant bacterial genera while *Cladosporium, Exidiopsis*, *Mycena*, *Penicillium* and *Trechispora* were reported as the dominant fungal genera [108]. Sette et al. [109] used a minimal inhibitory concentration test (MIC) to investigate the endophytic filamentous fungi against different human pathogenic bacteria from coffee plants (*Coffea arabica* and *C. robusta*) in Pedreira, Mococa, and Pindorama counties in Brazil. The study showed that 17 endophytic fungi were able to inhibit at least one of the bacterial pathogens while *Guignardia* sp. (CBMAI 69), *Phomopsis* sp. (CBMAI 164), and *Trichoderma harzianum* (CBMAI 43) were able to inhibit four to five bacterial species. Furthermore, the study showed that *Aspergillus versicolor* (CBMAI 46), *Cladosporium* sp. (CBMAI 64 and CBMAI 66), *Fusarium oxysporum* (CBMAI 53), and *Glomerella* sp. (CBMAI 63) can inhibit all pathogenic bacteria [109]. The previous studies show that coffee-endophytic fungi are diverse and different groups of fungi are able to inhibit different fungal and bacterial pathogens [58,59,60,61,62,63,108,109]. When comparing those results with our study, different endophytic fungi isolated in our study, such as *Arthrinium* sp., *Biscogniauxia* sp., *Daldinia* sp., *Diaporthe* sp. and *Nigrospora* sp., can also inhibit the growth of pathogens but they are not the same fungal genera that were reported in previous studies [58,59,60,61,62,63,108,109].

*Arthrinium* is known for its antifungal capacity [110]. The species *A. aureum* and *A. phaeospermum* have the potential to inhibit the growth of *Fusarium oxysporum* and *F. niveum* and thus can be applied in biological controls [111]. Our isolate *Arthrinium* sp. (XCE-10) showed an inhibition rate of 64.89% against the growth of *Penicillium digitatum*.

*Biscogniauxia* sp. (O-811) isolated from the fresh fruiting bodies of wild mushrooms acts against the rice blast disease *Magnaporthe oryzae* by producing an inhibitory compound [112], while our endophytic strain *Biscogniauxia* sp. (PTE-7) can inhibit the growth of the fungal pathogens *Alternaria alternata* and *Penicillium digitatum* while also inhibiting the bacterial pathogen *Salmonella enterica* subsp. *enterica* with inhibition rates of 61.11%, 67.56%, and 60.42%, respectively.

*Daldinia* cf. *concentrica* prevented the development of molds on organic dried fruits and eliminated *Aspergillus niger* infection in peanuts by emitting volatile organic compounds [113]. The endophytic fungus *Daldinia eschscholtzii* BPEF73 of black pepper showed nematocidal activity by producing metabolites [114]. Six *Daldinia* isolates (T5E-1-3, T5E-3, AKE-11, P4E-1, AKE-12, and ME-9) in our study showed good antagonistic abilities against all four pathogens tested. The isolate ME-9 inhibited *Penicillium digitatum* with the highest inhibition rate of 74.67%.

Studies have shown that some endophytic species belonging to the genus *Diaporthe* can produce metabolites that protect the host from infection or act as a biological control for disease. For example, the endophytic fungus *Diaporthe phaseolorum* isolated from tomatoes can produce 16 different compounds, and it has an inhibitory effect on the growth of *Xanthomonas vesicatoria,* which causes bacterial spot disease in tomatoes [115]. Dhakshinamoorthy et al. [116] showed that the endophytic fungus *Diaporthe caatingaensis* from *Buchanania axillaris* leaves can produce the bioactive metabolite Camptothecin (CPT). While our endophyte *Diaporthe* sp. ME-7 has inhibitory effects on the growth of the fungal pathogens *Alternaria alternata* (63.43%) and *Penicillium digitatum* (64.89%), it did not show any apparent inhibitory effects on bacterial pathogens.

*Nigrospora oryzae* was isolated as an endophytic fungus from the leaves of *Coccinia grandis* and its secondary metabolites can be used as drugs to control diabetes. They also exhibit strong antifungal activities against the plant pathogen *Cladosporium cladosporioides* [117]. Our four *Nigrospora* isolates T5E-13, T5E-7, XCE-7, and XCE-25 showed antagonistic abilities for all three pathogens. The best isolate among all of these was XCE-7 which showed the highest inhibition rate of 71.76% against *Alternaria alternata*.

Our study is the first report on coffee-leaf-endophytic fungal diversity in China. We also tested the antagonistic abilities of endophytic fungi present in coffee leaves against major bacterial and fungal pathogens. Two isolates (*Nigrospora* sp. XCE-7 and *Daldinia* sp. ME-9) showed antagonism against the growth of *Alternaria alternata,* with an inhibition rate of over 70%. Three isolates (*Daldinia* sp. ME-9, *Nigrospora* sp. T5E-7, and *Daldinia* sp. T5E1-3) displayed antagonism against the growth of *Penicillium digitatum,* with an inhibition rate of over 70%. It is necessary to carry out further research on secondary metabolites and identification of the four coffee endophytes ME-9, T5E-1-3, T5E-7, and XCE-7 to the species level.

## Figures and Tables

**Table 1:** Information on the host species and sample collection sites.

Host (Varieties)	Coffee Plantation Site in Pu’er City	Location (Latitude; Longitude; Altitude)	Abbreviation	Collection Date
*Coffea arabica* (Catimor)	Xiao Ao Zi coffee plantation (小凹子咖啡庄园)	22.6618° N; 100.9584° E; 1066 m	XCE	21 December 2020
	Qi Xiang coffee plantation (奇象咖啡庄园)	22.7040° N; 101.3462° E; 900 m	QCE	22 December 2020
	Mo Jiang Jing Gong coffee plantation (墨江晶工咖啡庄园)	23.2543° N; 101.7390° E; 1100 m	ME	23 December 2020
*Coffea arabica* (Aika)	Xiang Yuan Shu He coffee plantation (香橼树河咖啡庄园)	22.7541° N; 101.2869° E; 1006 m	AKE	22 December 2020
*Coffea arabica* (P4)			P4E	22 December 2020
*Coffea arabica* (PT)			PTE	22 December 2020
*Coffea arabica* (T5175)			T5E	22 December 2020
*Coffea arabica* (T8667)			T8E	22 December 2020
*Coffea arabica* (Yellow Bourbon)	Xiao Ao Zi coffee plantation (小凹子咖啡庄园)	22.6618° N; 100.9584° E; 1066 m	XYE	21 December 2020

**Table 2:** Records of coffee leaf fungal endophytes.

Class	Genus	AKE	ME	P4E	PTE	QCE	T5E	T8E	XCE	XYE	Total
Dothideomycetes	*Cladosporium*	-	1	-	-	-	-	-	1	-	2
	*Phyllosticta*	2	2	1	1	2	2	-	1	5	16
	*Pyrenochaetopsis*	-	-	-	-	-	1	-	-	-	1
	*Shiraia*	-	-	-	-	1	-	-	-	-	1
Sordariomycetes	*Annulohypoxylon*	1	-	1	1	-	-	1	-	-	4
	*Arthrinium*	-	-	-	-	-	-	-	2	3	5
	*Biscogniauxia*	1	-	-	1	-	-	-	-	-	2
	*Cladorrhinum*	1	-	1	1	-	-	2	-	-	5
	*Colletotrichum*	8	15	7	5	20	6	8	17	14	100
	*Daldinia*	5	1	4	3	4	3	4	-	1	25
	*Diaporthe*	-	2	1	-	-	-	-	-	-	3
	*Fusarium*	-	-	-	-	1	-	-	-	-	1
	*Hypoxylon*	-	-	-	1	-	-	2	1	1	5
	*Kretzschmaria*	-	-	-	1	-	-	-	-	-	1
	*Naviculispora*	-	-	-	-	-	1	-	-	-	1
	*Nemania*	-	-	-	-	-	-	-	-	2	2
	*Neurospora*	-	-	1	-	-	-	-	-	-	1
	*Nigrospora*	-	-	1	-	-	3	1	2	-	7
	*Nodulisporium*	-	-	-	-	-	-	2	-	-	2
	*Pestalotiopsis*	-	-	-	-	-	1	-	-	-	1
	*Xylaria*	6	-	5	4	1	3	6	1	3	29
-	Unknown	2	-	1	5	2	4	6	1	-	21
**Total**	**21**	**26**	**21**	**23**	**23**	**31**	**24**	**32**	**26**	**29**	**235**

**Table 3:** Information of the pathogens obtained from CGMCC.

Pathogen Group	Species Name	Strain Number	Major Disease
Bacteria	*Pseudomonas syringae*	CGMCC 1.3333	Canker, halo blight of bean [70]
	*Salmonella enterica* subsp. *enterica*	CGMCC 1.10603	Food-borne illness [72]
Fungi	*Alternaria alternata*	CGMCC 3.15535	Black spots [64]
	*Penicillium digitatum*	CGMCC 3.15410	Fruit post-harvest diseases [66]

**Table 4:** Records and diversity of fungal endophytes associated with coffee hosts from different population sites. R = individuals/records, S = number of species, G = number of genera, d = Margalef diversity, H’ = Shannon diversity index, J’ = Pielou evenness index, CR = colonization rate, IF = isolation frequency.

Isolate Name	Leaf Pieces	R	S	G	S/G Ratio	d	H’	J’	CR (%)	IF (%)
AKE	30	26	12	7	1.71	3.9901	2.4382	0.9506	86.67%	11.06%
ME	30	21	11	5	2.20	3.2846	2.2524	0.9393	70.00%	8.94%
P4E	30	23	18	9	2.00	5.7407	2.8944	0.9830	76.67%	9.79%
PTE	30	23	14	9	1.56	4.4650	2.5218	0.9312	76.67%	9.79%
QCE	30	31	16	6	2.67	4.3681	2.5246	0.9106	103.33%	13.19%
T5E	30	24	15	8	1.88	4.7199	2.6582	0.9587	80.00%	10.21%
T8E	30	32	15	9	1.67	4.3281	2.6205	0.9452	106.67%	13.62%
XCE	30	26	13	7	1.86	3.9901	2.2125	0.8384	86.67%	11.06%
XYE	30	29	13	7	1.86	3.5637	2.3303	0.9085	96.67%	12.34%

**Table 5:** Results of the dual-culture assay of 61 selected endophytes against the four pathogens. The type of fungal interaction for each isolate against each pathogen and the percentage of growth inhibition (I%) ± standard deviation (SD) is indicated.

Isolate Code	Species Name	Strain Number	GenBank Accession Number (ITS)	Percent Inhibition (PI) of 10 Days ± SD			
				*Penicillium digitatum* CGMCC 3.15410	*Alternaria alternata* CGMCC 3.15535	*Pseudomonas syringae* CGMCC 1.3333	*Salmonella enterica* subsp. *enterica* CGMCC 1.10603
AKE-1	*Xylaria* sp.	KUMCC 21-0349	ON100618	52.00 ± 3.56	43.98 ± 5.57	28.89 ± 9.88	41.67 ± 8.68
AKE-3	*Xylaria* sp.	KUMCC 21-0350	ON100619	52.00 ± 14.22	44.44 ± 5.14	0.00 ± 0.00	39.58 ± 8.68
AKE-6	*Colletotrichum* sp.	KUMCC 21-0351	ON100620	59.11 ± 1.58	44.44 ± 1.29	24.44 ± 2.47	43.75 ± 26.04
AKE-7	*Cladorrhinum* sp.	KUMCC 21-0352	ON100621	52.00 ± 15.41	43.52 ± 0.43	22.22 ± 9.88	52.08 ± 8.68
AKE-11	*Daldinia* sp.	KUMCC 21-0353	ON100622	67.56 ± 5.14	60.00 ± 2.04	31.11 ± 9.88	39.58 ± 8.68
AKE-12	*Daldinia* sp.	KUMCC 21-0354	ON100623	64.00 ± 4.74	60.19 ± 0.43	53.33 ± 29.63	54.17 ± 8.68
AKE-19	*Annulohypoxylon* sp.	KUMCC 21-0355	ON100624	53.33 ± 1.19	43.98 ± 3.00	20.00 ± 29.63	43.75 ± 26.04
ME-7	*Diaporthe* sp.	KUMCC 21-0364	ON100633	64.89 ± 7.51	63.43 ± 0.43	37.78 ± 9.88	40.63 ± 8.68
ME-8	*Colletotrichum* sp.	KUMCC 21-0365	ON100634	50.22 ± 0.40	45.83 ± 1.29	28.89 ± 39.51	41.67 ± 8.68
ME-9	*Daldinia* sp.	KUMCC 21-0366	ON100635	74.67 ± 8.30	71.30 ± 0.43	60.00 ± 7.41	50.00 ± 6.51
ME-10	*Colletotrichum* sp.	KUMCC 21-0367	ON100636	51.56 ± 5.14	46.30 ± 3.00	31.11 ± 2.47	41.67 ± 8.68
ME-16	*Colletotrichum* sp.	KUMCC 21-0368	ON100637	57.78 ± 0.40	44.44 ± 1.29	26.67 ± 29.63	29.17 ± 8.68
PTE-3	*Hypoxylon* sp.	KUMCC 21-0356	ON100625	58.67 ± 3.56	56.94 ± 5.14	4.44 ± 9.88	39.58 ± 8.68
PTE-6	*Xylaria* sp.	KUMCC 21-0357	ON100626	50.22 ± 0.40	37.50 ± 9.00	0.00 ± 0.00	45.83 ± 34.72
PTE-7	*Biscogniauxia* sp.	KUMCC 21-0358	ON100627	67.56 ± 0.40	61.11 ± 1.29	53.33 ± 0.00	60.42 ±8.68
PTE-9	*Xylaria* sp.	KUMCC 21-0359	ON100628	50.22 ± 0.40	43.06 ± 1.29	35.56 ± 9.88	25.00 ± 26.04
PTE-10	*Annulohypoxylon* sp.	KUMCC 21-0360	ON100629	57.78 ± 2.77	58.33 ± 0.00	31.11 ± 17.28	39.58 ± 34.72
PTE-12	*Phyllosticta* sp.	KUMCC 21-0361	ON100630	46.67 ± 18.96	44.44 ± 1.29	28.89 ± 17.28	37.50 ± 6.51
PTE-18	*Kretzschmaria* sp.	KUMCC 21-0362	ON100631	47.56 ± 0.40	37.96 ± 3.00	0.00 ± 0.00	39.58 ± 8.68
PTE-19	*Xylaria* sp.	KUMCC 21-0363	ON100632	47.56 ± 0.40	41.20 ± 5.57	0.00 ± 0.00	39.58 ± 8.68
P4E-1	*Daldinia* sp.	KUMCC 21-0380	ON072525	64.89 ± 6.32	54.63 ± 1.71	46.67 ± 29.63	41.67 ± 8.68
P4E-2	*Xylaria* sp.	KUMCC 21-0381	ON072526	46.67 ± 1.19	29.63 ± 3.00	33.33 ± 0.00	37.50 ± 0.00
P4E-4	*Daldinia* sp.	KUMCC 21-0382	ON072527	47.56 ± 0.40	53.70 ± 3.00	4.44 ± 39.51	47.92 ± 8.68
P4E-8-2	*Xylaria* sp.	KUMCC 21-0383	ON072528	49.78 ± 0.40	30.56 ± 1.29	8.89 ± 69.14	39.58 ± 8.68
P4E-9	*Phyllosticta* sp.	KUMCC 21-0384	ON072529	44.89 ± 2.77	30.56 ± 1.29	33.33 ± 0.00	41.67 ± 8.68
P4E-11	*Colletotrichum* sp.	KUMCC 21-0385	ON072530	47.56 ± 2.77	26.39 ± 1.29	22.22 ± 39.51	37.50 ± 0.00
P4E-16	*Colletotrichum* sp.	KUMCC 21-0386	ON072531	40.89 ± 0.40	32.87 ± 3.00	33.33 ± 29.63	39.58 ± 8.68
P4E-18	*Nigrospora* sp.	KUMCC 21-0387	ON072532	43.56 ± 0.40	29.17 ± 1.29	28.89 ± 69.14	45.83 ± 8.68
P4E-23	*Cladorrhinum* sp.	KUMCC 21-0388	ON072533	43.56 ± 0.40	28.24 ± 3.00	31.11 ± 32.10	43.75 ± 0.00
P4E-26	*Diaporthe* sp.	KUMCC 21-0389	ON072534	46.22 ± 0.40	34.72 ± 1.29	31.11 ± 9.88	41.67 ±8.68
QCE-13	*Xylaria* sp.	KUMCC 21-0397	ON072542	37.33 ± 1.19	24.07 ± 3.00	2.22 ± 9.88	25.00 ± 26.04
QCE-14	*Daldinia* sp.	KUMCC 21-0398	ON072543	44.89 ± 0.40	53.70 ± 1.71	24.44 ± 9.88	41.67 ± 8.68
QCE-17	*Colletotrichum* sp.	KUMCC 21-0399	ON072544	44.89 ± 1.58	34.26 ± 3.00	6.67 ± 29.63	45.83 ± 34.72
QCE-20-2	*Phyllosticta* sp.	KUMCC 21-0400	ON072545	44.44 ± 1.58	30.56 ± 1.29	0.00 ± 0.00	41.67 ± 8.68
QCE-24	*Colletotrichum* sp.	KUMCC 21-0401	ON072546	55.56 ± 2.77	44.44 ± 1.29	22.22 ± 69.14	47.92 ± 8.68
QCE-26	*Colletotrichum* sp.	KUMCC 21-0402	ON072547	46.67 ± 1.19	34.72 ± 1.29	0.00 ± 0.00	39.58 ± 8.68
T5E-1-3	*Daldinia* sp.	KUMCC 21-0369	ON100638	70.67 ± 4.74	63.43 ± 3.00	55.56 ± 9.88	60.42 ± 8.68
T5E-3	*Daldinia* sp.	KUMCC 21-0370	ON100639	68.00 ± 1.19	68.52 ± 0.43	21.11 ± 17.28	39.58 ± 8.68
T5E-7	*Nigrospora* sp.	KUMCC 21-0371	ON100640	72.00 ± 1.19	65.74 ± 3.00	13.33 ± 29.63	41.67 ± 6.51
T5E-13	*Nigrospora* sp.	KUMCC 21-0372	ON100641	65.78 ± 2.77	48.61 ± 1.29	27.78 ± 17.28	52.08 ± 8.68
T5E-19	*Phyllosticta* sp.	KUMCC 21-0373	ON100642	51.56 ± 2.77	36.11 ± 5.14	24.44 ± 9.88	35.42 ± 8.68
T8E-1	*Daldinia* sp.	KUMCC 21-0374	ON100643	52.89 ± 11.06	55.56 ± 5.14	46.67 ± 29.63	5.00 ± 0.00
T8E-4	*Nodulisporium* sp.	KUMCC 21-0375	ON100644	58.22 ± 2.77	45.37 ± 3.00	35.56 ± 9.88	43.75 ± 26.4
T8E-8-1	*Xylaria* sp.	KUMCC 21-0376	ON100645	45.78 ± 2.77	43.98 ± 3.00	13.33 ± 29.63	41.67 ± 8.68
T8E-11-1	*Xylaria* sp.	KUMCC 21-0377	ON100646	43.56 ± 2.77	45.37 ± 0.43	35.56 ± 9.88	35.42 ± 8.68
T8E-12	*Xylaria* sp.	KUMCC 21-0378	ON100647	44.44 ± 5.14	43.06 ± 1.29	28.89 ± 39.51	41.67 ± 15.19
T8E-22	*Nigrospora* sp.	KUMCC 21-0379	ON100648	53.33 ± 1.19	43.98 ± 0.43	2.22 ± 9.88	43.75 ± 26.04
XCE-2	*Cladosporium* sp.	KUMCC 21-0403	ON072548	42.67 ± 1.19	32.41 ± 0.43	8.89 ± 39.51	39.58 ± 8.68
XCE-4	*Colletotrichum* sp.	KUMCC 21-0404	ON072549	47.56 ± 2.77	37.50 ± 1.29	4.44 ± 9.88	37.50 ± 26.04
XCE-7	*Nigrospora* sp.	KUMCC 21-0405	ON072550	68.00 ± 1.19	71.76 ± 0.43	61.11± 32.10	56.25 ± 0.00
XCE-8	*Xylaria* sp.	KUMCC 21-0406	ON072551	46.67 ± 1.19	30.56 ± 1.29	40.00 ± 29.63	29.17 ± 8.68
XCE-10	*Arthrinium* sp.	KUMCC 21-0407	ON072552	64.90 ± 2.77	57.87 ± 3.00	28.89 ± 39.51	35.42 ± 15.19
XCE-25	*Nigrospora* sp.	KUMCC 21-0408	ON072553	67.56 ± 6.32	62.50 ± 1.29	15.56 ± 9.88	45.83 ± 34.82
XCE-26	*Lasiosphaeriaceae* sp.	KUMCC 21-0409	ON072554	48.44 ± 0.40	34.26 ± 3.00	26.67 ± 0.00	33.33 ± 8.68
XYE-4	*Colletotrichum* sp.	KUMCC 21-0390	ON072535	42.22 ± 1.58	32.87 ± 1.71	15.56 ± 39.51	41.67 ± 8.68
XYE-9	*Daldinia* sp.	KUMCC 21-0391	ON072536	47.56 ± 0.40	31.48 ± 0.43	22.22 ± 9.88	41.67 ± 8.68
XYE-13	*Cladorrhinum* sp.	KUMCC 21-0392	ON072537	44.44 ± 0.40	29.17 ± 1.29	46.67 ± 29.63	56.25 ± 0.00
XYE-14	*Hypoxylon* sp.	KUMCC 21-0393	ON072538	44.44 ± 2.77	34.72 ± 1.29	33.33 ± 0.00	35.42 ± 8.68
XYE-16-2	*Phyllosticta* sp.	KUMCC 21-0394	ON072539	46.22 ± 0.40	31.02 ± 5.57	2.22 ± 9.88	39.58 ± 8.68
XYE-18	*Xylaria* sp.	KUMCC 21-0395	ON072540	38.22 ± 5.14	31.94 ± 1.29	33.33 ± 0.00	39.58 ± 8.68
XYE-19-2	*Arthrinium* sp.	KUMCC 21-0396	ON072541	44.44 ± 2.77	34.72 ± 1.29	15.56 ± 9.88	27.08 ± 8.68

## Data Availability

Not applicable.
